# Progress in Surgery

**Published:** 1897-11-13

**Authors:** 


### PROGRESS IN SURGERY.

### THE SURGICAL TREATMENT OF FOCAL EPILEPSY.

Since our last *résumé* of the recent literature on the surgical aspect of epilepsy, a number of valuable papers have appeared both in America and Germany. The general tendency of these has been to confirm the opinion, which has for some time been growing, that the success of operations undertaken for the relief of this most distressing condition is much less than was hoped for some years ago.

The opinions of Sachs and Gerster are of special interest.[1] These writers have made an unbiassed study of the various surgical procedures undertaken for the relief of partial epilepsies, including under this term all cases in which "a circumscribed cortical lesion is the cause of the epilepsy." Nineteen cases are critically analysed. The writers are not inclined to take too pessimistic a view of surgical intervention, because they believe that, in view of the gravity of the disease, even an improvement, or an occasional cure, will justify the surgeon in performing these operations, which are not of themselves necessarily dangerous.

The writers insist that no case of epilepsy can be said to be cured unless the patient has been entirely free from all attacks for a period of at least a year. They suggest that three years would be a still safer limit. At the same time they draw attention to the fact that the recurrence of fits a few days after operation is not to brand a case as a failure, because improvement often sets in after considerable periods, and remains permanently. Much importance is attached to the administration of bromides, chloral, opium, &c., for some time after these operations.

In publishing statistics of a series of cases the writers hold that it is of comparatively little value simply to state the ultimate result. To be of real value the cases should be so reported as to bring out the reasons for success or failure as the case may be. This they have done with their 19 cases. Three of the patients were cured; 5 were improved, but not cured; 11 were unimproved by operation. The points brought out by an analysis of the records of these cases are, that the sooner the operation is performed after the onset of epileptic seizures the better is the prognosis. In the cases where recovery was not complete, such reasons as too early return to hard work and abuse of alcohol are given as possible explanations of the failure. In all the cases of complete failure long intervals had elapsed between the onset of the disease and operation. The excision of cortical areas appears only to be useful in cases treated very early. In cases of old standing it presents no advantages. The paper is summed up in the following conclusions: (1) Surgical interference is advisable in those cases of partial epilepsy in which not more than one year, or at the utmost two years, have elapsed since the traumatic injury or the beginning of the disease which has given rise to the convulsive seizures. (2) In cases of depression or other injury of the skull, surgical interference is warranted, even though a number of years have elapsed; but the prospect of recovery is brighter the shorter the period of time since the injury. (3) Simple trephining may prove sufficient in a number of cases, and particularly in those in which there is an injury to the skull, or in which a cystic condition is the main cause of the epilepsy. (4) Excision of cortical tissue is advisable if the epilepsy has lasted but a short time, and if the symptoms point to a strictly circumscribed focus of disease. (5) Since such cortical lesions are often of a microscopical character, excision should be practised even if the tissue appears to be perfectly normal at the time of operation; but the greatest caution should be exercised in order to make sure that the proper area is removed. (6) Surgical interference for the cure of epilepsy associated with infantile cerebral palsies may be attempted, particularly if too long an interval has not elapsed since the beginning of the palsy. (7) In cases of epilepsy of long standing, in which there is in all probability a widespread degeneration of the association fibres, every surgical procedure is useless.

Professor Nancrede[2] emphasises the importance of publishing the "end-results" of cases operated upon. He comments on four cases of his own. The first case illustrates the clinical fact that certain forms of epilepsy may be due to irritation of nerves by cicatrices either of bone or soft parts. The patient had a cicatrix on the left side of the scalp, pressure on which induced a fit, commencing on the same side as the injury, and therefore not of cortical origin. Another surgeon treated the case by trephining in the region of this scar, but relief was only temporary. As a sequel to this operation, the scalp, meninges, and brain all became fused in a very dense cicatrix, with the result that a true cortical epilepsy was established, now affecting the opposite side primarily. Nancrede operated for this condition by excising the new-formed scar along with the thumb centre. For two and a half years the patient was free of fits, but recurrence then took place, doubtless from further scar formation. The second case was one in which fits had occurred for five years before an operation was performed. The centres for the angle of the mouth and tongue on the left side were removed. The patient was absolutely free from fits for over three years, but now has fits at long intervals. In the third, apparent recovery followed an operation in which nothing more than exposing the brain was done. Nancrede looks upon the improvement as a coincidence, although there are various feasible explanations. The fourth case was operated upon for fits which followed one another with such extreme rapidity that the patient was for some time before operation in a status epilepticus. Two months later, while working in the fields, he became acutely maniacal, and his old attacks returned. The author has been reluctantly forced to the following conclusions: (1) Removal of the discharging lesion in cortical and Jacksonian epilepsy can only be regarded as palliative; the operation scar becomes a new source of irritation; (2) the earlier the operation is done after the disease becomes fully established the longer will the immunity last, and it is possible that if trephining is done very early it may in some cases be curative; (3) the operation is not dangerous, and often gives a long period of immunity, and the fits after relapse are less severe and less frequent; (4) when the paroxysms are practically continuous, the discharging lesion should be removed to save life; (5) a long period of inactivity is imperative after these operations; (6) general treatment is not to be neglected after operations, because the operation only removes one factor in the condition.

Still another writer—Braun[3]—has recently analysed a series of cases, 22 in number, where the cortical centre from which the attacks apparently started was removed. Fourteen of these are claimed as "completely successful," but in five only has the immunity lasted for longer than a year. This writer does not think that the cortical centre should be removed, until a less severe operation, involving only the skull and membranes, has proved futile. Collins,[4] on the other hand, emphasises the importance of excising the cortical centre, in view of the fact that he, and others, have found distinct pathological conditions present in parts so removed. The lesions found have been: (1) Chronic meningo-encephalitis; (2) marked obliteration in the blood vessels of the pia and cortex, associated with formation of new capillaries, which have undergone hyaline degeneration; (3) slow degenerative changes in the ganglion cells, with slight neuroglia hyperplasia; (4) softened areas at the junction of grey and white matter; (5) replacement of these softened areas by true neuroglia tissue; (6) multiple hæmorrhages throughout the cortex. In the discussion which followed this paper M. A. Starr stated that out of 24 cases he had operated upon there was not a single cure, recurrence taking place in from one to three and a half years. All the cases were carefully selected as suitable for operation. W. W. Keen also is unable to cite a case of his known to be cured, although he is more hopeful of the operation than Starr. He thinks three years a reasonable time at which to consider a case as definitely cured. He considers the palliation, or even the temporary freedom from fits, sufficient justification for the operation in many cases. Brill (*ibid*) is inclined to blame the physicians for the unsatisfactory nature of the statistics of this operation, because they do not sufficiently discriminate between suitable and unsuitable cases. Of 14 cases in which he has seen craniotomy performed for epilepsy, in only two was there any benefit. Both of these were cases of depressed fracture. The only argument in favour of operation that he could urge is admittedly unjustifiable, namely, to induce euthanasia.

Cases illustrative of the various points brought out in these papers referred to are reported by Mills Roberts,[5] L. G. Guthrie,[6] A. E. Morison,[7] and Higgins.[8]

[1] Amer. Jour. Med. Sciences, Oct., 1896. [2] Annals of Surg., Aug., 1896. [3] Centralbl. fur Chir., No. 44, 1896. [4] Jour. of Nervous and Mental Disease, Oct., 1896. [5] Lancet, Oct. 17, 1896. [6] Brit. Med. Jour., Dec. 12, 1896. [7] Brit. Med. Jour., Oct. 17, 1896. [8] Med. Rec., Feb. 6, 1897.

